# Certified Registered Nurse Anesthetists’ occupational exposure to inhalational anesthetic agents: a survey of anesthetic gas safety

**DOI:** 10.1186/s12871-022-01896-y

**Published:** 2022-12-03

**Authors:** Trent Masselink, Jan Hardinger, Carrie Bowman-Dalley, Crystal O’Guinn, Kumudhini Hendrix, Nancy Crowell, Ladan Eshkevari

**Affiliations:** Georgetown University Medical Center, Washington, DC, USA

**Keywords:** Anesthesia, Anesthetic gas, Safety, Environmental exposure, Short-term Effects

## Abstract

**Background:**

Anesthetic gases have been known to cause damage when inhaled over long periods of time. Modern safety measures have been put in place to reduce the risk to anesthesia providers, however there is continued lack of information on providers experiencing short term effects (lethargy, fatigue, headache, slowed cognitive ability, nausea, and mucosal irritation) thereby leading to long-term sequalae (sister chromatid exchanges, micronuclei, chromosomal aberrations, and comet assays).

**Method:**

A thirteen item, multiple choice survey was sent to 3,000 anesthesia providers, of which 463 completed the survey. A Chi-square test of independence was used to determine the association between gas exposure and participant self-reported symptoms. A Spearman’s Correlation test was also utilized to interpret this data since both frequency of smelling gas and frequency of symptoms were ordinal variables for which Spearman’s *rho* correlation was the appropriate measure of association.

**Results:**

The major findings were that as the frequency of smelling anesthetic gas increased, so too did the frequency of self-reported headaches and fatigue. *Spearman’s rho* = .148 and .092. *P* value = .002 and .049, respectively.

**Conclusion:**

There have been many efforts to decrease the risk of exposure of anesthesia providers to anesthetic gases. While there is a decrease in reported exposures, indications of possible long-term effects remain a concern in anesthesia providers. Potential implications of exposure could lead to chromosomal aberrations, sister chromatid exchanges, comet assays, spontaneous abortions, and genotoxic effects.

**Supplementary Information:**

The online version contains supplementary material available at 10.1186/s12871-022-01896-y.

## Background

The purpose of this study was to evaluate the incidence of self-reported exposure to anesthetic gases and determine the impact of exposure with regards to related symptoms, such as lethargy, fatigue, headache, slowed cognitive ability, nausea, and mucosal irritation. Anesthetic gases have been used for over a hundred years and are a mainstay of anesthesia practice [1]. Among the most commonly used of these halogenated agents are sevoflurane, isoflurane, and desflurane. Halothane and enflurane are also used, but less frequently. These drugs are administered in the operating room (OR) by having patients breathe them in and are eliminated when patients exhale them out. The Occupational Safety and Health Administration (OSHA) and the National Institute for Occupational Safety and Health (NIOSH) have set limits on the levels of anesthetic gas in which healthcare personnel can be exposed to in the OR [1]. Current limitations are defined as a maximum exposure of 25 parts per million (ppm) of nitrous oxide and two ppm of inhalational agents [1–3].

Detrimental side effects of anesthetic gases can include lethargy and fatigue from short-term exposure. Long‐term exposure may be linked to spontaneous abortion, congenital abnormalities, and genotoxic damage [1]. Current research on exposure to anesthetic gases focuses on the physician and staff nurse population. Certified Registered Nurse Anesthetists (CRNAs) give around fifty million anesthetics a year and are in direct contact with patients receiving inhaled agents [4]. This makes them particularly susceptible to these side effects because of their close proximity to patients and to the machines used for volatile gas administration during each of these anesthetics given. Thus, in studies of occupational anesthetic gas exposure, this subset of the OR team deserves further investigation as to their exposure levels, the nature of their exposure, and current precautionary practices.

In several landmark studies fatigue was a common symptom reported by anesthesia personnel working in ORs, as was nausea [5, 6]. Another study examined those in the OR for more than 20 h a week and found almost 40% of subjects reported headaches, which they attributed to anesthetic gas exposure (*p* < *0.01*) [7]. When surveying 557 exposed OR nurses and technicians, a significantly higher number of headaches were reported by the exposed group (*p* < *0.01*) with 17% compared to 3% of the control group [1, 8]. In another study, healthy volunteers were exposed to 500 ppm nitrous oxide versus 500 ppm nitrous oxide with 15 ppm enflurane to determine cognitive ability impairment. A statistically significant (*p* < *0.05*) difference in the majority of their cognitive tests was noted in both groups at their respective exposure levels. The nitrous oxide and enflurane group experiences more cognitive ability impairment [9]. This study was repeated with halothane substituted for enflurane compared to nitrous alone, and again statistically significant (*p* < *0.05*) slowed cognitive abilities were observed [6]. In a study where subjects were asked to rate symptoms on a self-reported survey, 30% of subjects attributed symptoms such as rhinitis, to anesthetic gas exposure [7].

Chronic exposure can also lead to detrimental long-term effects. In a meta-analysis of 17 studies performed in ORs, levels of anesthetic gases were found to be significantly (*p* < *0.05*) above regulation levels where genotoxic damage can be seen [10]. In a survey study of anesthetists in the United Kingdom who had experienced spontaneous abortions and congenital abnormalities, the researchers found a significant increase (*p* < *0.025*) among female anesthetists compared to a control group of female non-anesthesia providers [11]. A similar study examined 60 OR personnel that included surgeons, nurses, and technicians that were exposed to inhalational agents on a 6-h time-weighted average [12]. In this study, prolonged exposure to volatile agents was correlated to spontaneous abortions as well as congenital abnormalities [2]. Another study conducted in Ontario hospitals found a statistically significant (*p* < *0.05*) increase in both spontaneous abortions and congenital abnormalities in a group of employees exposed to inhalational agents compared to a control group [3]. The American Society of Anesthesiologists (ASA) distributed a questionnaire to over 10,000 exposed OR personnel. It too found statistically significant (*p* < *0.01*) increased rates of spontaneous abortion and congenital abnormalities among exposed women compared to the general population [13].

Genotoxic effects occur when exposure to certain environmental substances causes damage to the genetic material coded within cells. This can lead to increased risk of diseases, similar to exposure to radiation [10]. A group of researchers performed a systematic review of articles on genotoxicity related to anesthetic gas exposure, which included exposure to nitrous oxide, halothane, isoflurane, desflurane, sevoflurane, and enflurane. All of the studies included in the review demonstrated statistically significant (*p* < *0.05*) increases in all genotoxic markers examined [10].

Because exposure to these inhalation agents can cause the aforementioned effects, two methods to reduce gas exposure have been implemented: air turnover systems and waste gas scavenging systems. Air turnover systems use sophisticated ventilation to exchange the air in the OR to decrease OR pollution [1, 14–16]. Whereas, waste gas scavenging systems remove excess gas in the anesthesia circuit [16, 17]; therefore, potentially reducing these genotoxic effects.

## Methods

This study was approved by Georgetown University Institutional Review Board GU-IRB #00002013, and was conducted using a correlational design that evaluated the self-reported incidence and self-reported symptoms of anesthetic gas exposure. The researchers explored percentages of reported anesthetic gas exposure among CRNAs with particular attention to environmental influence. Furthermore, the association between exposure and the self-reported symptoms of lethargy, fatigue, headache, slowed cognitive ability, and mucosal irritation were also examined.

The sample included CRNAs who are actively practicing in the United States. To obtain a representative sample, the American Association of Nurse Anesthetists (AANA) Research Department carried out a random selection of CRNA participants from among the association’s approximately 36,800 active, voting members. Eligibility for participation required that respondents be active CRNA members of the AANA who had not opted out of mass email communication. Exclusion criteria included: CRNAs who did not meet the inclusion criteria, student registered nurse anesthetists, and inactive, honorary, and graduate AANA members. The study was completely voluntary, and participation took place via response to an emailed survey.

Per the G*Power® Sample Size Calculator (Additional file 1: Appendix C), a sample size of *N* = 307 CRNAs was needed from a population of 3,000 (the AANA allows a maximum of 3,000 email addresses to be surveyed). In order to achieve a power of 0.80 with an alpha of 0.05 and a small to medium effect size of w = 0.2, adjusted for finite samples without replacement, using a chi-squared test with degrees of freedom = 6. The data collection tool was sent to a random sample, via the AANA Research Department to the maximum 3,000 survey addresses allowable. The final number of participants was *N* = 463.

The data collection tool was titled Self-Reported Effects to Anesthetic Gases (SREAG) and was designed by the researchers (Additional file 1: Appendix A). Due to the lack of evidence to support the validity and effectiveness of the newly designed research tool, five experts reviewed the data collection tool for relevance and content. The expert reviewers were chosen based on their academic, and/or research and practice backgrounds in anesthesia, and basic sciences. Once reviewed, the tool was delivered as an electronic survey using SurveyMonkey©. Approximately seven days before the survey deadline, the AANA Research Department’s survey system delivered an email to remind participants to complete the data collection tool.

A cover letter was included which identified the research questions being investigated as well as the contact information for the researcher and for Georgetown University’s IRB. The letter assured participants as to the confidentiality and anonymity of their responses in addition to explaining the process of consent. In this case, the submission of the survey implied consent to participate in the research study and was completely voluntary. Participants were informed that there were no risks or direct benefits associated with their participation and that they were free to withdraw at any time during the survey. No personally identifiable information was collected about survey participants. The AANA Research Department then organized the data into an Excel spreadsheet, which was stripped of email identifiers and contained no identifying information in the responses. This data was emailed to the researcher to be analyzed.

The survey tool consisted of thirteen multiple-choice questions, which were divided into three sections. The first section contained demographic questions, including subject age, gender, and employment status. The second section focused on self-reported exposure to anesthetic gases and the incidence of symptoms experienced by the respondents. Questions related to length of practice and nature of the practice, type of cases [e.g., Laryngeal Mask Airways (LMAs), pediatric, or Total Intravenous Anesthesia (TIVA)], were evaluated on a Likert scale. The questions that related to the frequency of self-reported exposure aimed to identify the percentage of cases that had a non-invasive airway, usually pediatric patients or those with a supraglottic airway (SGA), versus those patients with a definitive airway with an endotracheal tube. These questions also identified how many times per day the CRNA self-reported noticing the odor of anesthesia gas and what percent of their practice used anesthetic gases versus employing a TIVA technique. The third section contains five questions. These were also in the format of a Likert scale with subjects rating self-reported symptoms of exposure as less, same, and more comparing exposure days. Subjects were asked to rate their symptoms of lethargy, fatigue, headache, slowed cognitive ability, and mucosal irritation on days when anesthesia gas was used compared to the days when a TIVA technique was performed.

### Statistical analysis

Excel-formatted data were imported into and analyzed using IBM SPSS 27, a statistical analysis program. Data were categorical and nominal in nature for the demographic section of the survey, and primarily categorical in nature for the second and third sections of the survey. Chi-square analyses were used to interpret the data with respect to the research questions posed by the study. A Chi-square test of independence was used to determine the association between gas exposure and symptoms. *Spearman’s Rho* correlation was then used to determine the association between the frequency of smelling anesthetic gas and the frequency of self-reported symptoms.

## Results

A total of 463 surveys were initiated by the surveyed respondents. However, a few respondents did not answer all the questions in the survey. Therefore, each variable had a slightly different *n*.

Of the *n* = 463 respondents, 44.9% were 50–59 years old, 39.3% were 60 years or older, while only 15.8% of respondents were 40–49 years old. Of the *n* = 461, the majority of survey respondents (74.2%) reported more than 20 years of CRNA experience, 23.6% reported 16–20 years of experience, and only 2.2% reported 11–15 years of CRNA experience. One-hundred and eighty-three (39.6%) of respondents identified as being male and 279 (60.4%) identified as being female, with 76.5% reporting that they worked full-time as a CRNA, whereas 18.1% reported part-time working status, and 5.4% reported having both full-time and part-time CRNA positions.

The majority, 352 (76.5%) of the respondents reported that they smelled anesthetic gases at least 0–3 times during an average workday, and 82 (17.8%) reported smelling anesthetic gases 4–6 times per day, 15 (3.3%) reporting smelling anesthetic gases 7–9 times a day, and 11 (2.4%) reported smelling anesthetic more than 10 times per day.

To further ascertain information on anesthetic gas exposure, answers to survey questions were analyzed in relation to specific anesthetic techniques. While using an LMA, almost half, (45.8%) of respondents reported smelling anesthetic gases 0–30% of the time, with nearly another half, 199 (43.2%) of respondents reporting smelling anesthetic gases 31–60% of the time. Of note, 51 (11.1%) of respondents reported the frequency of smelling gases while using an LMA at 61–100% of the time during an average workday.

In performing pediatric anesthetics, a large majority, 430 (93.3%) of respondents reported smelling anesthetic gases 0–30% of the time, while 25 (5.4%) of respondents reported smelling gases 31–60% of the time, with the remaining few (1.3%) of respondents reported smelling gases 61–100% of the time.

Additionally, almost a third (28.3%) of the study participants reported that 0–30% of their cases utilized anesthetic gases over TIVA, with another third (27.6%) of respondents reporting that 31–60% of their practices included using anesthetic gases over TIVA, while most of the respondents, 204 (44.1%) reported that 61–100% of their anesthetic practice included using an anesthetic gas over TIVA approach.

A Spearman’s correlation test was performed to determine the relationship between the frequency of smelling anesthetic gas and the specific anesthetic technique used (LMA, pediatric, gas vs. TIVA). As shown in Table 1, there were no significant correlations between frequency of smelling gas and percent of time using LMA, performing pediatric cases, or using anesthetic gas vs. TIVA approach (Table 1).

**Table 1:** Spearman’s correlations between anesthetic practice technique and reports of smelling gas

Anesthetic Technique	Smelling Gas	*P*
	*Spearman’s rho*	
LMA cases	.055	.24
Pediatric cases	.068	.14
Anesthetic gas vs. TIVA	.064	.17

**Fig. 1:**
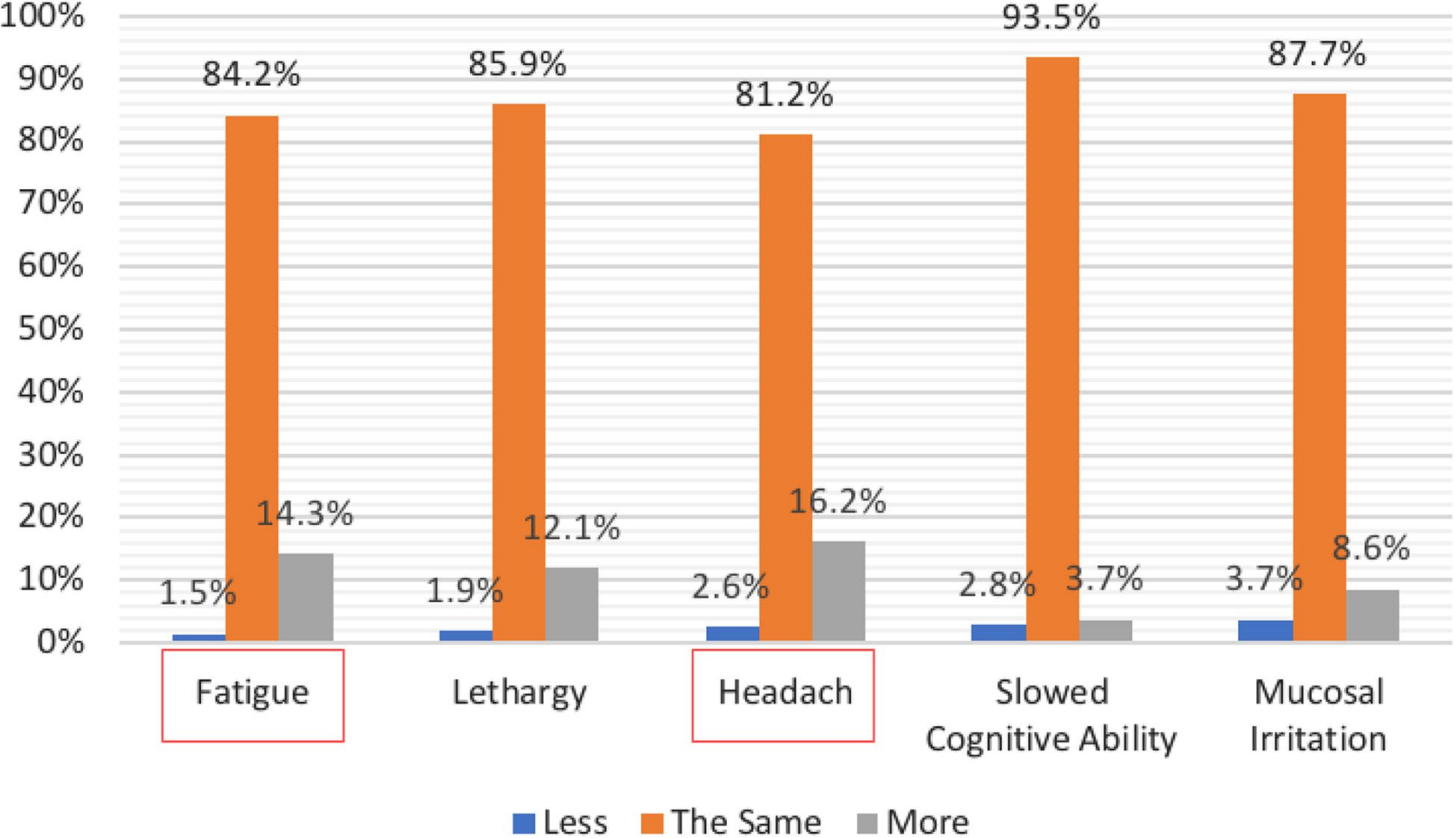
Respondent’s report on frequency of smelling anesthetic gases during an average workday and frequency of self-reported symptoms during those exposures. The Y-axis is the percent of respondents and the X-axis is the self-report symptoms. The frequency of symptoms is color coded: “less” (blue), “the same” (orange), and “more” (gray). The two circled items were significantly correlated with frequency of smelling gas (see Table 2)

Next, answers to survey questions were analyzed to determine the relationship between anesthetic gas exposure and a series of self-reported symptoms. The self-reported symptoms were chosen because the literature suggests that these symptoms are associated with anesthetic gas exposure. To gather this information, respondents were asked, “when using volatile agents on an average day compared to days without, how would you rate your: fatigue, lethargy, headache, slowed cognition, and mucosal irritation”? Respondents were also asked to specify if they experienced these symptoms “less”, “more”, or “the same” when using anesthetic gas vs. not during an average workday. There were positive, statistically significant correlations between frequency of smelling gas and frequency of fatigue and headache (Fig. 1).

## Discussion

Exposure to inhalation gases has been well documented as a potentially detrimental occurrence, frequently reported by anesthesia providers [2, 3, 18, 19]. Once exposed, there are a several self-reported symptoms that have the potential to negatively impact the health and wellness of the anesthesia provider [12, 20]. The purpose of this study was to evaluate the self-reported incidence of anesthetic gas exposure by CRNAs and to further examine the self-reported symptoms associated with said exposure.

The self-reported symptoms examined were lethargy, fatigue, headache, slowed cognitive ability, and mucosal irritation.

Our respondents were objectively older compared to AANA demographics with over 80% of respondents being older than 50 years of age, and a majority of them (74.2%) reported more than 20 years of CRNA experience. Of the 460 respondents, the majority of CRNAs reported that they smell anesthetic gases 0–3 times per average workday (76.5), while the remaining 23.5% reported smelling anesthetic gas more than 3 times per day (Fig. 1). These results are consistent with several reports that anesthetic gases are often found in high concentrations in the OR environment. For example, in a reported meta-analysis of 17 studies, levels of anesthetic gases were found to be above regulation levels where genotoxic damage can be seen [10].

Additionally, it was reported in several studies that levels of nitrous oxide, sevoflurane, and isoflurane, respectively, were found to be above OHSA and NIOSH standards [2, 3, 15]. These reports, as well as additional studies [6, 9, 12, 21–24] suggest that levels of anesthetic gases in the OR can be high enough to be detected by anesthesia providers. Indeed, our survey data confirmed reports of anesthetic gas exposure by the study participants.

The priority of our study was to determine the level of self-reported symptoms related to anesthetic gas exposure, and to assess potential short term side effects of anesthetic gas exposure [12, 20]. The common symptoms reported by anesthesia providers exposed to anesthetic gases reviewed in the literature were lethargy, fatigue, headache, slowed cognitive ability, nausea, and mucosal irritation [1, 6, 7, 12, 20, 23]. These symptoms are particularly concerning in an anesthesia provider, where they could compromise the ability of the provider to provide high quality, safe anesthetic care.

A positive, and statistically significant relationship (*p* = 0.049 and *p* = 0.002) existed between the frequency of smelling anesthetic gas and the frequency of the self-reported symptoms of fatigue and headache respectfully (Table 2). In multiple studies highlighted in the literature review [1, 5, 6, 20], fatigue was one of the most common self-reported symptoms in association to anesthetic gas exposure.

**Table 2:** Correlations between frequency of smelling gas and frequency of self-reported symptoms

Self-Reported Symptoms	Smelling Gas	*P*
	*Spearman’s rho*	
Fatigue (*n* = 462)	.092	.049
Lethargy (*n* = 462)	.089	.06
Headache (*n* = 458)	.148	.002
Slowed Cognitive Ability (*n* = 460)	.039	.40
Mucosal Irritation (*n* = 463)	.075	.11

Our findings support this information as 14.3% of 462 respondents answered they experienced more fatigue when exposed to anesthetic gas. Prior research distinctly differentiates fatigue and lethargy, where fatigue is a lack of physical energy and lethargy is loss of motivational force; therefore, the two were separated from each other in our deployed survey [19, 20].

A landmark study by Vaisman et al. revealed provider fatigue in the operating room can lead to underperformance by exposed personnel and potential patient harm [5, 6]. Therefore, this suggests the participant’s self-report of fatigue may have serious clinical implications. Future research may still be needed to confirm that self-reported fatigue is a problem. Perhaps, fatigue could be confirmed objectively by measuring the concentration of gas in the room. A validated, objective test could then be used to measure provider fatigue. Futhermore, more strictly defined safety limits, improved gas scavenging methods, or refined surveillance is needed to ensure that this potentially detrimental, self-reported symptom is not experienced by CRNA’s. These findings suggest that it may be necessary to reduce anesthetic gas exposure in its entirety.

Of the survey respondents, 28 people reported experiencing more headaches when exposed to anesthetic gas. A research study, exploring how healthy the operating room environment is; including anesthetic gas levels, safety protocols, and gas turn over systems, examined patients to determine the most common same-day symptoms of anesthetic gas exposure, headache was found to be in the top five [25]. Our findings are supported by multiple studies in the literature [7, 20, 23, 25], including a survey of OR personnel who were exposed to anesthetic gases for more than 20 h per week. Almost 40% of subjects reported headaches attributed to anesthetic gas exposure (*p* < 0.01). These results may be inconsistent with our findings because it is possible that our subjects had less than 20 h per week of exposure. The older age of our surveyed participates may have also skewed headache results.

Our results showed a positive, statistically significant correlation between the exposure of anesthetic gas and self-reported symptoms of fatigue and headache; however, in contrast to reviewed literature, there was no correlation found in our survey of CRNAs between anesthetic gas exposure and the self-reported symptoms of lethargy, slowed cognitive ability, and mucosal irritation. Rational and possible limitations to these findings will be discussed.

Incidental Findings show that of the 463 respondents who answered the demographic questions, 44.9% were 50–59 years old, 39.3% were 60 years or older, while only 15.8% of respondents were 40–49 years old. With over half of the respondents older than 50 years old, researchers questioned whether or not provider age would impact olfactory sensation and the ability to smell, as detailed in the literature [9]. Incidentally, our findings showed no relationship between age and frequency of smelling gas, Spearman’s rho = -0.06, *P* = 0.24.

There are several limitations to this study that, should be considered in future research. Although the survey was sent out to the maximum allowed survey respondents, only 463 responded. The response rate of this study was only 6.5%; however, average response rates to AANA surveys have been 5–7% in the past [4].

This study is also limited by population sample. It is important to note that there are other anesthesia providers who administer anesthetic gases on a daily basis, including physician anesthesiologist and anesthesia assistants. This survey was only implemented in the AANA survey database and only asked CRNAs about anesthetic gas exposure and self-reported symptoms; therefore, the survey is limited to a single profession.

Perhaps in the future, a similar survey could be deployed to all anesthesia providers in all capacities. This would not only increase the total population sample size but would provide a more diverse sample. A significant limitation that may have impacted data was the timing in which the AANA survey was released. The survey was deployed in April, 2020 and was made available to prospective participants for 40 days. Unfortunately, this was at the start of the COVID19 pandemic that significantly impacted the United States. Perhaps prospective respondents were more focused on personal health and national crisis rather than responding to a survey evaluating CRNA anesthetic gas exposure. While it could not have been predicted, asking what mask was worn and seeing if there was a correlation between N95’s and standard surgical masks due to their widespread use during this time could have affected outcomes.

Additionally, the survey relies on self-reports and perception of symptoms. This subjective measurement tool can be a fairly unreliable source of data due to the possible of recall and bias influences responses.

Potential follow up studies that employ objective measurements of gas concentrations in the OR may improve the detection of gas related symptoms. For example, headache scales the day of exposure and cognitive tests could be used instead of a survey.

The findings from this study may suggest that several changes may be needed to reduce anesthetic gas exposure by the CRNA and subsequent self-reported symptoms. Once the CRNA is exposed, it can impact their health and wellness and can therefore affect patient care [12, 20]. Improvement in anesthetic gas scavenging and operating room turnover techniques may be necessary to reduce the CRNAs exposure to the inhalation gas. Current gas limitations are defined as a maximum exposure of 25 ppm of nitrous oxide and 2 ppm of halogenated agents [1–3]. This regulation has been in place for over 40 years (2), but some studies have found negative effects at exposure levels below these guidelines [10].

Additionally, facility-based education programs or professional societies and associations could be useful in informing anesthesia providers on methods to reduce anesthetic gas exposure. Sources of gas waste include patient exhalation, leakage from around masks, leakage from poor fittings on a machine, spillage during refilling, or spillage from accidental disconnection of the patient breathing circuit [1]. Educating CRNAs on these high areas of exposure and methods to reduce them could decrease the symptoms associated with inadvertent anesthetic gas exposure.

## Conclusion

Exposure to inhalation gases by anesthesia providers has been well documented as potentially detrimental [2, 3, 18, 19]. Once reportedly exposed, there are several self-reported symptoms that have the potential to negatively impact the health and wellness of the CRNA, and most importantly, affect patient outcomes [12, 20]. It was discovered that of the 5 self-reported symptoms of fatigue, lethargy, headache, slowed cognitive ability, and mucosal irritation, exposure to anesthetic gas was significantly associated with headache and fatigue. These symptoms, experienced by the CRNA, could negatively impact patient outcomes. This may necessitate the need for more robust methods and procedures used to reduce anesthetic gas exposure reported by the certified nurse anesthetist population [4].

## Supplementary Information

**Additional file 1: Appendix A.** Survey. **Appendix B.** χ² tests - Goodness-of-fit tests: Contingency tables. **Appendix C.** Cover Letter/Informed Consent.

## Data Availability

The datasets used and/or analyzed during the current study are available from the corresponding authors on reasonable request.

## Abbreviations

OR: Operating Room
OSHA: The Occupational Safety and Health Administration
NIOSH: National Institute for Occupational Safety and Health
PPM: Parts per million
CRNA: Certified Registered Nurse Anesthetist
LMA: Laryngeal mask airway
TIVA: Total intravenous anesthesia
SCE: Sister chromatid exchange
MN: Micronuclei
CA: Comet assays
AANA: American Association of Nurse Anesthetists
SRNA: Student Registered Nurse Anesthetist
SREAG: Self-Reported Effects to Anesthetic Gases
SGA: Supraglottic airway
IRB: International Review Board
CITI: Collaborative Institutional Training Initiative
HIPAA: Health Insurance Portability and Accountability Act
