# Correction: Certified Registered Nurse Anesthetists’ occupational exposure to inhalational anesthetic agents: a survey of anesthetic gas safety

**DOI:** 10.1186/s12871-023-01980-x

**Published:** 2023-01-14

**Authors:** Trent Masselink, Jan Hardinger, Carrie Bowman-Dalley, Crystal O’Guin, Kumudhini Hendrix, Nancy Crowell, Ladan Eshkevari

**Affiliations:** Georgetown University Medical Center, Washington, DC USA

**Correction: BMC Anesthesiol 22, 375 (2022)**

**https://doi.org/10.1186/s12871-022-01896-y**

Following publication of the original article [1], the authors identified an error in the author name of Crystal O’Guin.

The incorrect author name is: Crystal O’Guinn

The correct author name is: Crystal O’Guin

The author group has been updated above and the original article [1] has been corrected.
