# Supplementary material for: Certified Registered Nurse Anesthetists’ occupational exposure to inhalational anesthetic agents: a survey of anesthetic gas safety
Source: BMC Anesthesiol. 2022 Dec 3;22:375. doi: 10.1186/s12871-022-01896-y (PMC9719248; doi:10.1186/s12871-022-01896-y)
Supplement: Supplementary file 1 — Additional file 1: Appendix A. Survey. Appendix B. χ² tests - Goodness-of-fit tests: Contingency tables. Appendix C. Cover Letter/Informed Consent. [file APPENDIX C Masselink Hardinger .pdf › APPENDIX C Masselink Hardinger .pdf]

APPENDIX C: Cover Letter/Informed Consent

Subject: You are invited to a research survey—OCCUPATIONAL EXPOSURE TO INHALATIONAL ANESTHETIC AGENTS.

Dear CRNAs,

You are invited to participate in a research study titled "OCCUPATIONAL EXPOSURE TO INHALATIONAL ANESTHETIC AGENTS." This study is being conducted by Trent Masselink, SRNA, at Georgetown University. The research questions guiding this study are: 1) What is the self-reported incidence of anesthetic gas exposure in the certified registered nurse anesthetist population? 2) What are self-reported symptoms related to anesthetic gas exposure in the certified registered nurse anesthetist population? This study is the primary researcher's doctoral research project and results may be submitted to peer reviewed journals to bring attention to the effects of anesthetic gas exposure in the OR.

Participation in this study is entirely voluntary at all times. You can choose not to participate or to leave the study at any time. Regardless of your decision, there will be no effect on your relationship with the researcher or any other consequences.

You are being asked to take part in this study because you are a CRNA who has practiced in the United States. If you agree to participate, you will be asked to fill out a single survey about your practice in relation to the frequency of anesthetic gas usage and your perception of symptoms possibly felt by the provider while using anesthetic gases. Survey questions are in multiple-choice format. This survey should take around 10 minutes to complete. The survey will be collected via SurveyMonkey upon submission via webpage application.

All of your responses to this survey will remain anonymous and cannot be linked to you in any way. No identifying information about you will be collected at any point during the study, and your survey will be identified only with a random number/letter sequence. Once you submit your completed survey, there will be no way to withdraw your responses from the study because the survey data contains no identifying information and cannot be traced back to your submission.

Study data will be kept in digital format via SurveyMonkey's secured database, and eventually Excel-formatted to the primary researcher's data analysis program. Access to digital SurveyMonkey data will be protected by data encryption and data exported will be password-secure. Only the primary researcher, Trent Masselink, will have access to the data.

There are no risks associated with this study. While you will not experience any direct benefits from participation, information collected in this study may benefit the profession of anesthesia in the future by creating a better understanding of the effects of anesthetic gas exposure. By determining the current levels of symptoms caused by anesthetic gas exposure, this study will assess and bring further awareness to this area of anesthesia practitioner workplace environmental risks.

If you have any questions regarding the survey or this research project in general, please contact the principal investigators, Trent Masselink, via email at tm1312@georgetown.edu and Jan Hardinger via email at jsh137@georgetown.edu or their faculty advisor, Dr. Ladan Eshkevari, via email at eshkevl@georgetown.edu. If you have any questions about your rights as a research participant, please contact the Georgetown University IRB at (202) 687-1506 or irboard@georgetown.edu.

By completing and submitting this survey you are indicating your consent to participate in the study. Your participation is greatly appreciated.

Trent Masselink, SRNA, Doctoral Candidate, Georgetown University

Jan Hardinger, SRNA, Doctoral Candidate, Georgetown University

Advisor Dr. Ladan Eshkevari, DNAP Program, Georgetown University
