# Supplementary material for: Certified Registered Nurse Anesthetists’ occupational exposure to inhalational anesthetic agents: a survey of anesthetic gas safety
Source: BMC Anesthesiol. 2022 Dec 3;22:375. doi: 10.1186/s12871-022-01896-y (PMC9719248; doi:10.1186/s12871-022-01896-y)
Supplement: Supplementary file 1 — Additional file 1: Appendix A. Survey. Appendix B. χ² tests - Goodness-of-fit tests: Contingency tables. Appendix C. Cover Letter/Informed Consent. [file 12871_2022_1896_MOESM1_ESM.zip › Masselink Hardinger BMC Appendices.docx]

**Appendix A**

Survey

1.     What is your age?

20-29

30-39

40-49

50-59

60+

2. How long have you been practicing?

0-5

6-10

11-15

16-20

20+

3.     What is your gender?

M

F

4.     Are you Full time, Part-time, or Both?

Full time

Part-time

Both

5.     During an average workday, how often would you say you smell an anesthetic gas?

0-3

4-6

7-9

10+

6. In your current practice, what percent of your practice is using LMAs?

0-30

31-60

61-100

7. In your current practice, what percent of your practice are pediatric cases?

0-30

31-60

61-100

8.     What percent of your practice is with Anesthetic gases versus a TIVA technique?

0-30

31-60

61-100

For Questions 9-13 when using volatile agents in an average day compared to days without, how would you rate your:

9.     FATIGUE? (extreme tiredness (i.e., lack of energy) and reduced functional capacity)

| Less | The Same | More |
| --- | --- | --- |
|  |  |  |

10.     LETHARGY? (a state of sluggishness, inactivity, and apathy)

| Less | The Same | More |
| --- | --- | --- |
|  |  |  |

11.     HEADACHE?

| Less | The Same | More |
| --- | --- | --- |
|  |  |  |

12.  SLOWED COGNITIVE ABILITY?

| Less | The Same | More |
| --- | --- | --- |
|  |  |  |

13.  MUCOSAL IRRITATION OF THE EYES, EARS, AND/OR NOSE?

| Less | The Same | More |
| --- | --- | --- |
|  |  |  |

**Appendix B**

χ² tests - Goodness-of-fit tests: Contingency tables

Analysis:	A priori: Compute required sample size

Input:	Effect size w                	=	0.2

α err prob                   	=	0.05

Power (1-β err prob)   =	0.8

Df                           	=	6

Output:	Noncentrality parameter λ    	=	13.6400000

Critical χ²                  	=	12.5915872

Total sample size          =	341

Actual power               =	0.8005383
